# YO-AFD: an improved YOLOv8-based deep learning approach for rapid and accurate apple flower detection

**DOI:** 10.3389/fpls.2025.1541266

**Published:** 2025-03-12

**Authors:** Dandan Wang, Huaibo Song, Bo Wang

**Affiliations:** ^1^ College of Communication and Information Engineering, Xi’an University of Science and Technology, Xi’an, China; ^2^ Xi’an Key Laboratory of Network Convergence Communication, Xi’an, China; ^3^ College of Mechanical and Electronic Engineering, Northwest A&F University, Xianyang, China; ^4^ Key Laboratory of Agricultural Internet of Things, Ministry of Agriculture and Rural Affairs, Xianyang, China; ^5^ Shaanxi Key Laboratory of Agricultural Information Perception and Intelligent Services, Xianyang, China; ^6^ School of Automation Science and Engineering, Faculty of Electronic and Information Engineering, Xi’an Jiaotong University, Xi’an, China

**Keywords:** apple flower, object detection, deep learning, YO-AFD, attention mechanism

## Abstract

The timely and accurate detection of apple flowers is crucial for assessing the growth status of fruit trees, predicting peak blooming dates, and early estimating apple yields. However, challenges such as variable lighting conditions, complex growth environments, occlusion of apple flowers, clustered flowers and significant morphological variations, impede precise detection. To overcome these challenges, an improved YO-AFD method based on YOLOv8 for apple flower detection was proposed. First, to enable adaptive focus on features across different scales, a new attention module, ISAT, which integrated the Inverted Residual Mobile Block (IRMB) with the Spatial and Channel Synergistic Attention (SCSA) module was designed. This module was then incorporated into the C2f module within the network’s neck, forming the C2f-IS module, to enhance the model’s ability to extract critical features and fuse features across scales. Additionally, to balance attention between simple and challenging targets, a regression loss function based on Focaler Intersection over Union (FIoU) was used for loss function calculation. Experimental results showed that the YO-AFD model accurately detected both simple and challenging apple flowers, including small, occluded, and morphologically diverse flowers. The YO-AFD model achieved an F1 score of 88.6%, mAP50 of 94.1%, and mAP50-95 of 55.3%, with a model size of 6.5 MB and an average detection speed of 5.3 ms per image. The proposed YO-AFD method outperforms five comparative models, demonstrating its effectiveness and accuracy in real-time apple flower detection. With its lightweight design and high accuracy, this method offers a promising solution for developing portable apple flower detection systems.

## 1 Introduction

The apple flowering stage is pivotal for apple tree growth. Precisely detecting and counting apple flowers during this phase aids growers in closely monitoring tree development. This allows for tailored formulation of fertilization, irrigation, and pest control strategies, thus enhancing orchard management efficiency and fruit quality. Furthermore, accurate flower detection is vital for forecasting peak flowering periods and early estimating yields. However, apple trees grow in intricate natural environments. Apple flowers, typically measuring 3-5 cm in diameter, are small and bloom at varying times, resulting in notable differences in individual flower phenotype. During blooming, apple flowers cluster together, with petals of different flowers overlapping each other, causing significant occlusion between them. Additionally, branches, leaves, insects, and other objects may occlude flowers, making precise detection using machine vision technology exceptionally challenging (Bhattarai et al., 2020; Sun et al., 2021; Mu et al., 2023; Zhou et al., 2024).

Researchers have explored fruit flower detection using computer vision technology (Zhou et al., 2017; Dias et al., 2018a; Zhao et al., 2020; Lim et al., 2020; Palacios et al., 2020; Estrada et al., 2024; Tan et al., 2024), achieving success in detecting flowers of various fruits such as citrus, peach, and apple (Dorj and Lee, 2012; Hočevar et al., 2014; Horton et al., 2017; Lin et al., 2020; Shang et al., 2024). Initially, studies relied on color features and threshold segmentation for detection. However, in uncontrolled orchard environments, adjusting threshold parameters became necessary due to changes in illumination conditions, backgrounds, and image acquisition angles. This dependence on parameter adjustments significantly affected detection accuracy, resulting in low reliability, poor stability, susceptibility to noise, and limited adaptability. Recently, with advancements in deep learning theory, researchers have turned to deep learning-based approaches for fruit flower detection. These approaches construct models with multiple hidden layers and leverage extensive datasets to automatically learn more useful features for flower detection. In contrast to traditional methods, deep learning-based approaches avoid the need for complex image pre-processing and tedious feature extraction, and effectively enhance detection accuracy and robustness. Dias et al. (2018b) employed a Convolutional Neural Network (CNN) for apple flower detection. Their approach involved utilizing the Clarifai CNN to extract features from apple flowers segmented by the simple linear iterative clustering (SLIC) superpixel algorithm, and applying a support vector machine (SVM) to ascertain the presence of apple flowers within the image. Experimental results demonstrated that the proposed CNN-based model facilitated precise flower detection, achieving optimal recall and precision rates approaching 80%. Remarkably, even when dealing with datasets dissimilar from the training data, the model maintained robust performance. Zhou et al. (2018) introduced a method for detecting the main organs of tomatoes utilizing a channel-wise group convolutional network. They chose a basic network structure and then augmented it by incorporating a channel-oriented grouped convolutional module with a Dropout layer and a full convolutional layer for organ detection. The method achieved an average precision of 96.5% and a recall of 77.4% in detecting tomato flowers. Deng et al. (2020) utilized Mask R-CNN (He et al., 2020), which based on ResNext-50 (Xie et al., 2017) and Feature Pyramid Network (FPN) (Lin et al., 2017), for the detection of citrus flowers in natural scenes. The average precision of the method in detecting citrus flowers was 36.3%, and the error in flower quantity calculation was 11.9%. The method served as a foundation for decision-making regarding flower quantity control. Wu et al. (2020) proposed a method centered on channel pruned YOLOv4 for detecting apple flowers. This method adeptly streamlined the model while preserving detection accuracy. The mean Average Precision (mAP) for detecting apple flowers reached 97.3%, with a detection speed of 72.3 Frames Per Second (FPS) and a model size of 12.5 MB. Tian et al. (2020) introduced a MASU R-CNN model, an advancement from the Mask Scoring R-CNN (Huang et al., 2019), tailored for the detection and segmentation of apple flowers. The authors innovatively fused the U-Net (Ronneberger et al., 2015) backbone with the MaskIoU head of Mask Scoring R-CNN to form a new MaskIoU head. This integration effectively enhanced the efficiency of feature utilization and facilitated the reuse of features by concatenating feature maps during the encoding and decoding processes. The experimental results revealed that the method achieved an F1 score of 95.9% and mAP of 59.4% on their dataset. Farjon et al. (2020) and Rahim and Mineno (2020) employed Faster R-CNN (Ren et al., 2017) for the detection of apple flowers and tomato flowers, respectively. Their researches demonstrated the efficiency of Faster R-CNN in fruit flower detection. Li et al. (2022) utilized YOLOv3 and YOLOv4 for detecting kiwifruit flowers and buds. Their comparative analysis revealed that YOLOv4 outperformed YOLOv3 in terms of detection performance. Wang et al. (2022) introduced an enhanced version of the YOLOv4 model termed YOLO-PEFL for precise detection of pear flowers in natural settings. The YOLO-PEFL model employed ShuffleNetv2 integrated with the Squeeze-and-Excitation Networks module as the backbone, replacing the original backbone network of YOLOv4. The experimental results showcased the effectiveness of the YOLO-PEFL model, achieving an average precision of 96.7% with an average detection speed of 27 ms. Notably, the model size of YOLO-PEFL was reduced by approximately 80% compared to YOLOv4, demonstrating significant efficiency improvements.

You Only Look Once (YOLO) tackles target detection by framing it as a regression problem, providing outputs that include the location and category of the detected target (Redmon and Farhadi, 2018; Bochkovskiy et al., 2020). As a one-stage detector, YOLO boasts rapid detection speeds and high accuracy, rendering it widely applicable in detecting various targets (Wang and He, 2021; Qi et al., 2022; Li et al., 2023, 2024). Compared with the previous versions of YOLO, YOLOv8 enhances the previous versions by further improving accuracy and flexibility, solidifying its position as the primary choice for tasks like object detection and image segmentation. Yang et al. (2023) proposed a novel LS-YOLOv8s model, which was based on the YOLOv8s deep learning algorithm and incorporated the LW-Swin Transformer module, for detecting and grading the ripeness of strawberries. Their innovation has led to improvements in both the accuracy and efficiency of detection. Wang et al. (2024) designed a normalization-based attention module named C2f-N, which was specifically crafted for residual feature learning, to replace the C2f block in the baseline YOLOv8 model. Their method achieved precise detection and segmentation of tomato targets.

The attention mechanism plays a pivotal role in deep learning by enabling models to prioritize essential information while disregarding irrelevant details. This mechanism effectively boosts the feature extraction capabilities of the model and enhances its ability to capture intricate feature information. Currently, attention mechanisms have been widely used in various object detection (Min et al., 2023; Zhu et al., 2024).

Based on the successful experience of previous researches, to address the challenge of detecting apple flowers under complex orchard, an improved YOLOv8 network, named YO-AFD, that fusing attention mechanism and YOLOv8 was proposed. The specific objectives are as follows: (1) A new attention module, ISAT, which integrated the Inverted Residual Mobile Block (IRMB) with the Spatial and Channel Synergistic Attention (SCSA) module was designed to enable adaptive attention to features across different scales. (2) A C2f-IS module, which incorporated ISAT into the C2f module within the network’s neck was developed to enhance the model’s ability to perceive targets of various sizes and morphologies. (3) Focaler Intersection over Union (FIoU) was used for loss calculation to analyze the impact of the sample distribution with various degrees of difficulty in bounding box regression, to further improve the performance in detection task. The paper is structured as follows: Section 2 presents the acquired dataset, and the methodology pipeline, including a detailed description of the deep neural network used for apple flower detection; Section 3 evaluates the performance of the proposed method, and Section 4 conducts the ablation experiment and discusses the results; Finally, Section 5 presents the conclusions and suggestions for future research.

## 2 Materials and methods

### 2.1 Dataset preparation

The images used in this research were taken within a commercial apple orchard located in Wuquan, Xianyang, Shaanxi, China. The variety of apple was Granny Smith. The specific image acquisition times were 9:00-11:30 a.m. and 2:30-5:00 p.m. on March 28 (sunny) and March 29 (cloudy) in 2021. To ensure the diversity of the image samples, images were captured under natural daylight conditions, encompassing both backlight and direct sunlight scenarios. The images were captured using an iPhone 11 Pro Max with a resolution of 3024 pixels×4032 pixels and saved in JPEG format.

**Figure 1:**
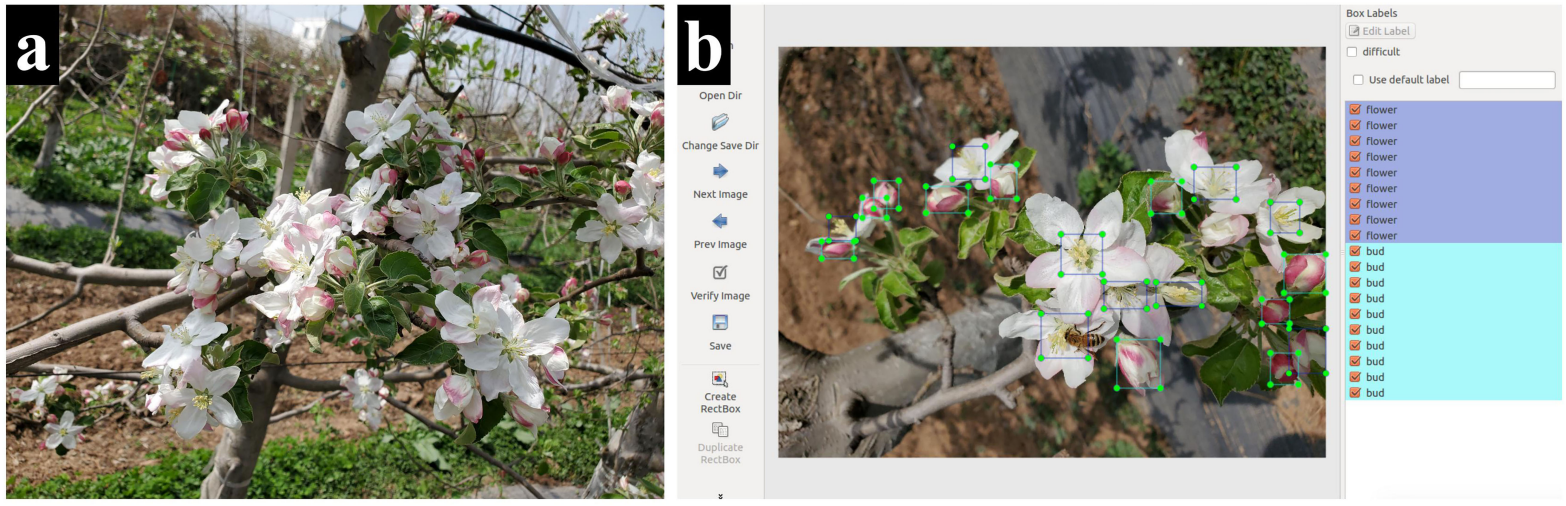
Example of apple flower image and annotations. **(a)** Example of apple flower image. **(b)** Example of apple flower image annotations.

In this study, a total of 2115 images of apple flowers were captured. To balance detail retention and algorithmic efficiency, the images were resized to 605 pixels × 807 pixels for the subsequent experiments. These images then underwent manual labeling using LabelImg. During the annotation process for apple flowers, the following cases should be taken into account (as shown in **Figure 1a**):

(1) Since apple flowers do not bloom simultaneously, there are notable differences in the individual morphology of each flower. Both buds and fully bloomed flowers coexist simultaneously, and the number of petals in fully bloomed flowers varies.
(2) Apple flowers tend to cluster together, resulting in the overlapping of petals from different flowers, which complicates the identification of individual flowers. Additionally, there is substantial overlap and occlusion between the flowers, further exacerbating the difficulties in distinguishing them.
(3) In an unstructured open environment, flowers are often occluded by branches, leaves, bees, and other objects, and the natural lighting conditions and lighting angles change over the time. Furthermore, both backlight and direct sunlight conditions are prevalent, leading to the formation of shadows cast by surrounding objects onto the surface of the flowers.

To ensure the accuracy of the subsequent established network model, stamen were annotated as the open flower. For apple flowers with invisible stamens, the entire flower was annotated. For unopened buds, the entire flower buds were annotated (as shown in **Figure 1b**).

A total of 43, 031 targets, including flowers and buds were labeled. After image annotation, the dataset was split into a training set (1, 270 images), a validation set (425 images) and a test set (420 images) with a ratio of approximately 6:2:2, for subsequent network training and parameter optimization. The detailed information of the image dataset used in this study is shown in **Table 1**.

**Table 1:** Detailed information of image set.

Number	Images	Flowers	Buds	Total Labeled
Training Set	1270	9394	20574	29968
Validation Set	425	2778	4075	6853
Test Set	420	2208	4002	6210
Total	2115	14380	28651	43031

### 2.2 Model construction

#### 2.2.1 Description of YO-AFD apple flower detection method

**Figure 2:**
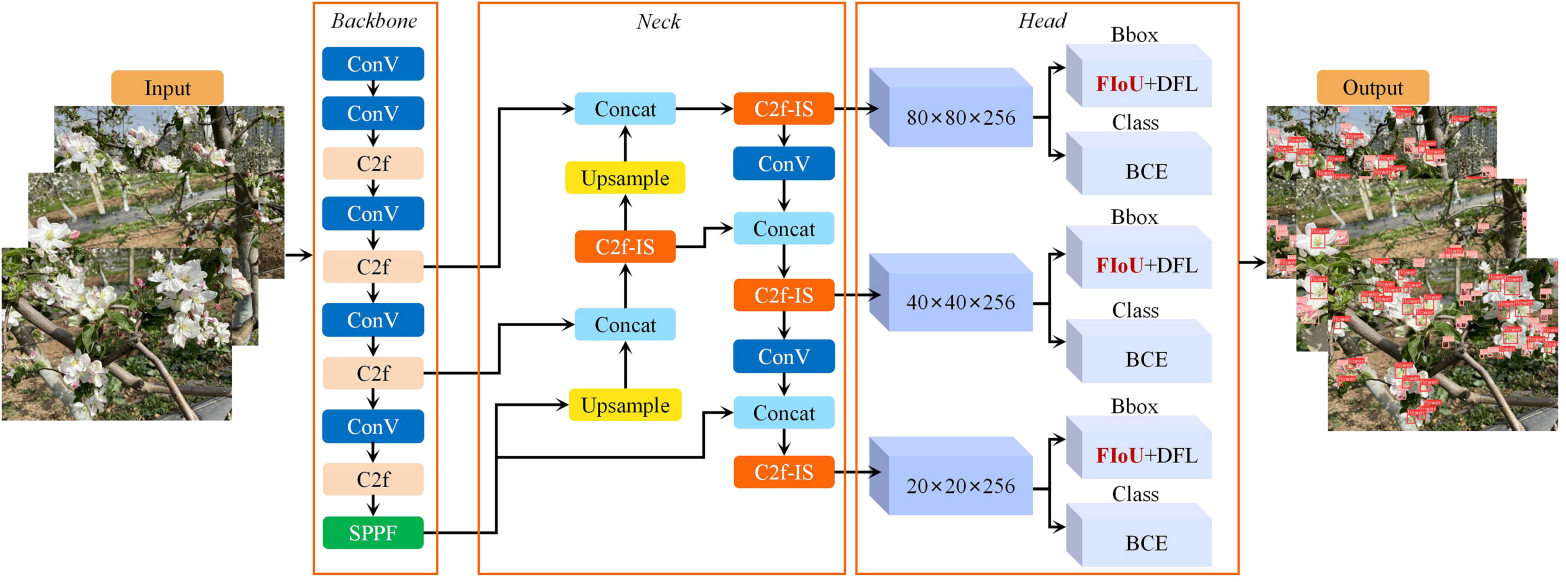
Structure of improved YO-AFD apple flower detection method.

The YOLO series, a one-stage deep learning model for object detection, has been widely adopted in the agricultural domain. YOLOv8, developed by Ultralytics in 2023, introduces significant advancements and optimizations over its predecessors, positioning it as the preferred solution for tasks such as object detection and image segmentation. Compared to other algorithms in the YOLO series, YOLOv8 achieves a balance between accuracy and processing speed, making it well-suited for applications that require fast detection while maintaining relatively high accuracy. This study aims to rapidly and accurately detect apple flowers in images captured from a natural orchard environment. Leveraging the strengths of YOLOv8, an enhanced method, termed YO-AFD, was specifically designed for apple flower detection. The architecture of YO-AFD is illustrated in **Figure 2**.

YO-AFD retains the same structural framework as the baseline YOLOv8n network, consisting of three primary components: Backbone, Neck, and Head. The backbone was used for feature extraction. To address the challenges posed by small and complex targets in natural orchards, the YO-AFD network incorporates several key improvements over the baseline YOLOv8n. Notably, an attention-based fusion feature learning module, C2f-IS, grounded in ISAT, was developed to enhance the model’s ability to extract and integrate apple flower features in the complex orchard environment. Additionally, the FIoU loss function, which replaced the CIoU loss function of the baseline YOLOv8n, was combined with the Distribution Focal Loss (DFL) as the regression function. These enhancements are intended to improve detection accuracy and robustness, especially in challenging orchard conditions. The details of the YO-AFD model will be further elaborated in the following subsections.

#### 2.2.2 C2f-IS attention fusion feature learning module

**Figure 3:**
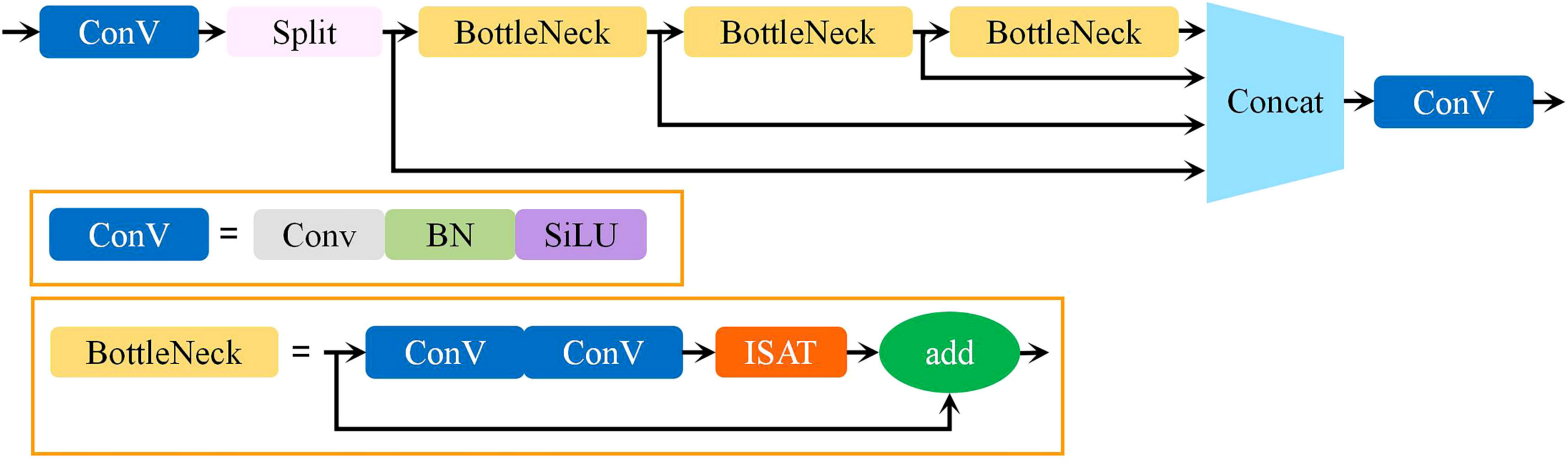
Structure of C2f-IS.

In this study, a novel attention fusion feature learning module based on the ISAT attention mechanism, termed C2f-IS, was proposed. The C2f-IS module, depicted in **Figure 3**, is integrated into the neck of the YO-AFD network, as shown in **Figure 2**. The ISAT module is a lightweight and efficient attention mechanism designed by integrating the Inverted Residual Mobile Block (IRMB) (Zhang et al., 2023) with the Spatial and Channel Synergistic Attention (SCSA) module (Si et al., 2024).

**Figure 4:**
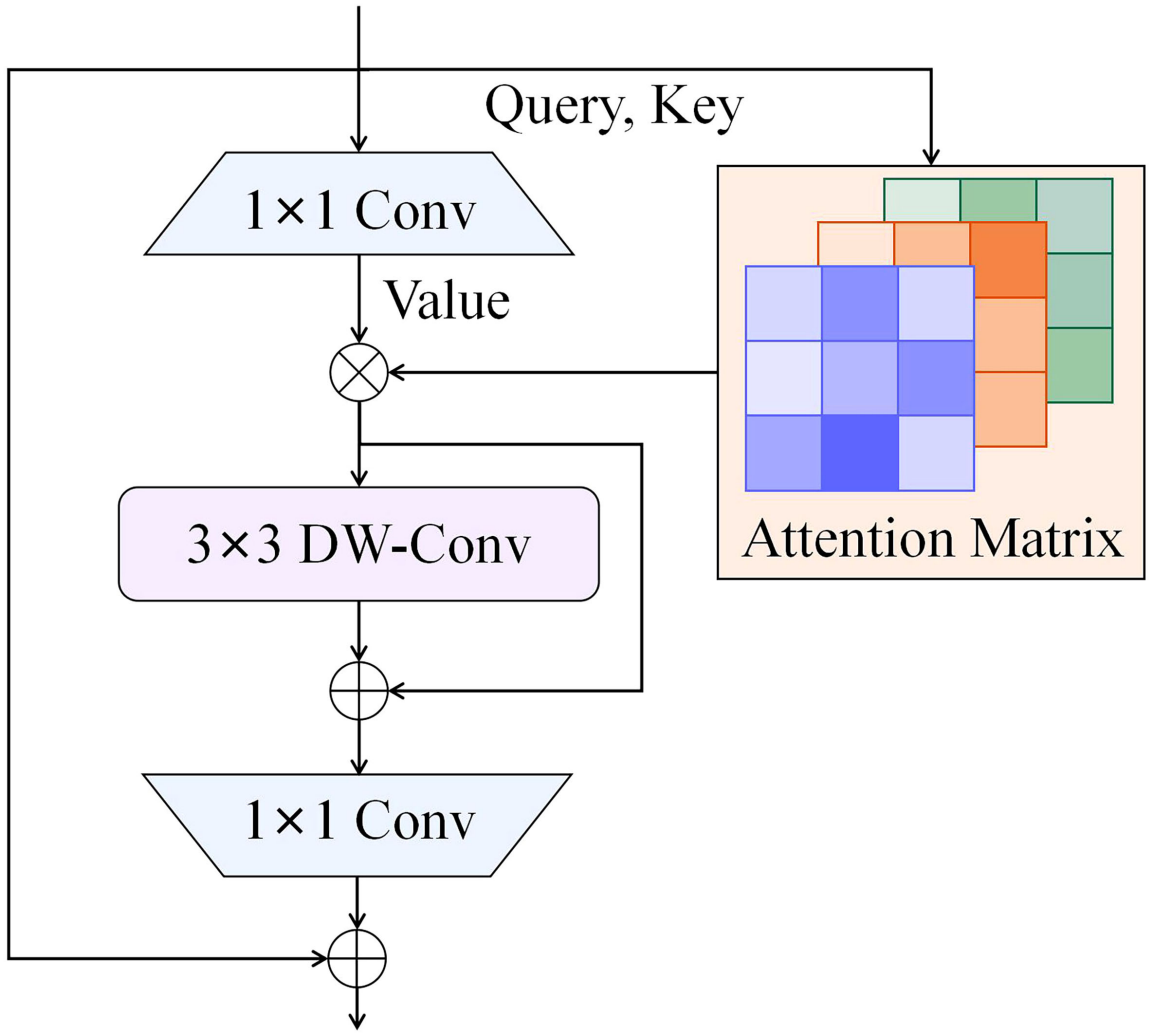
Paradigm of iRMB.

IRMB is a hybrid network block that combines the lightweight characteristics of convolutional neural networks with the dynamic processing capabilities of Transformer models, thus enhancing the ability to process long-range information. This design retains efficiency of model while effectively utilizing computational resources and achieving high accuracy. The paradigm of IRMB is shown in **Figure 4**. IRMB integrates Depth-Wise Convolution (DW-Conv) and Multi-Head Self Attention mechanisms. The 1×1 convolution within the structure is employed to compress and expand channel dimensions, thus enhancing computational efficiency. The 3×3 DW-Conv is used to capture spatial features, and attention mechanism is utilized to capture global dependencies among features. This configuration enables IRMB to consider the entire input space during feature extraction, thereby enhancing its ability to comprehend complex data patterns.

**Figure 5:**
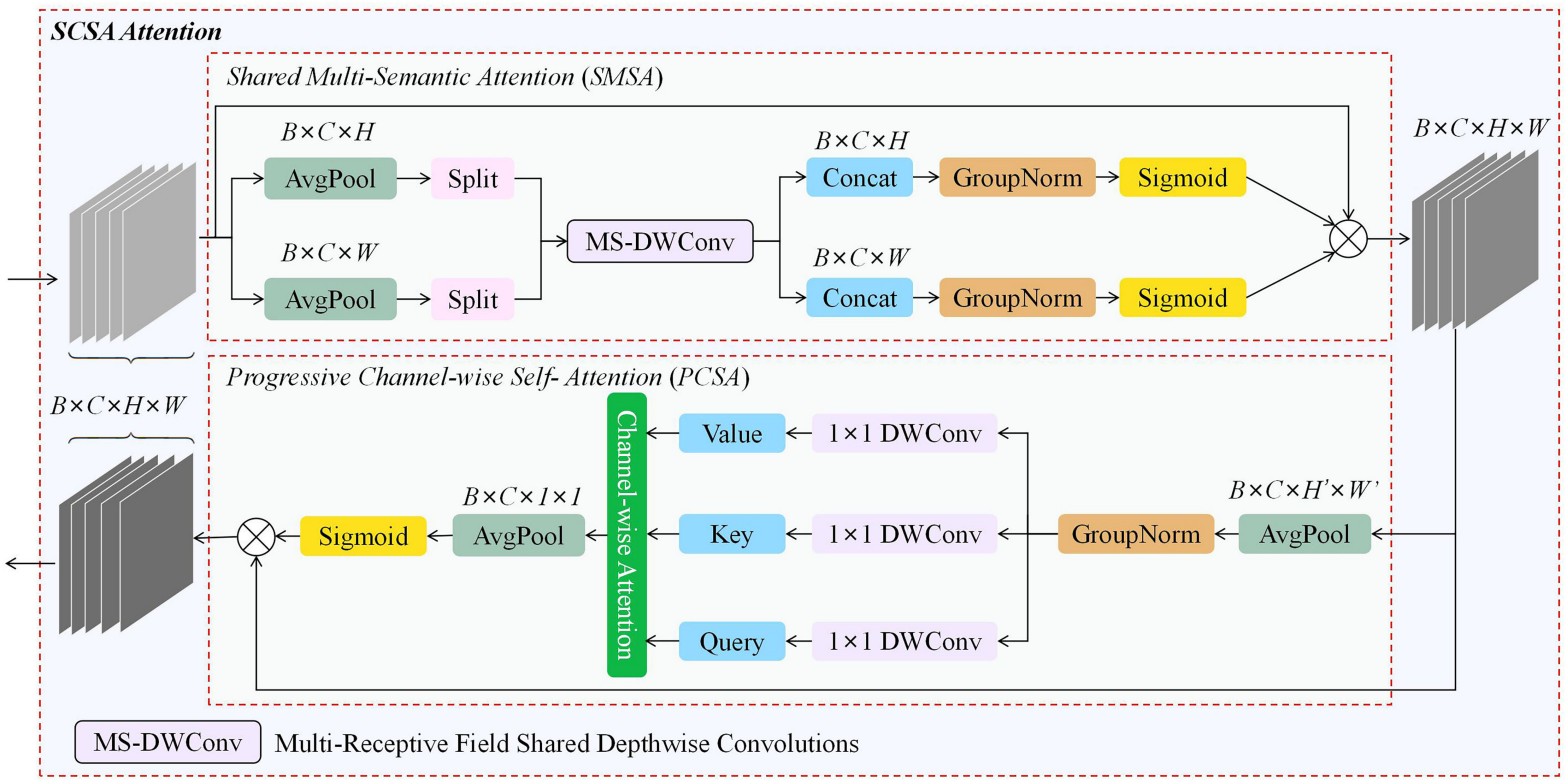
Structure of SCSA.

SCSA is a dual-component attention module consisting of two key parts: Shareable Multi-Semantic Spatial Attention (SMSA) and Progressive Channel-wise Self-Attention (PCSA). The structure of SCSA is shown in **Figure 5**. SMSA extracts rich semantic features and multi-level spatial information from multi-scale spatial inputs, providing multi semantic spatial priors for PCSA’s channel-wise self-attention. This enhances the representation of different semantic information. The robust feature interactions facilitated by the self-attention mechanism in PCSA help to mitigate the disparities in multi-semantic information across different sub-features within SMSA. Overall, SCSA demonstrates strong performance and good generalization ability in object detection tasks (Si et al., 2024).

**Figure 6:**
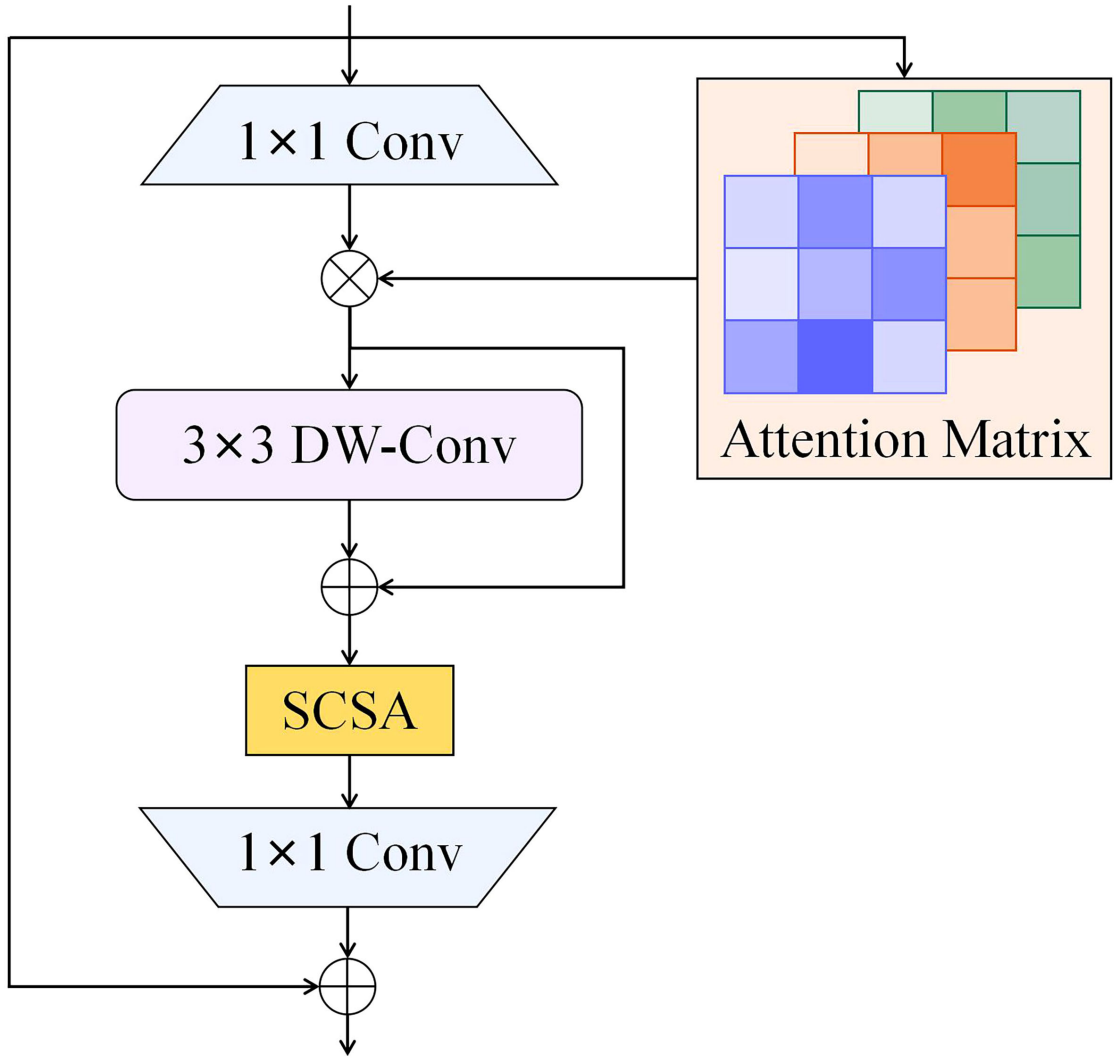
Paradigm of ISAT.

The IRMB improves feature processing by utilizing an inverted residual block, which allows for the effective capture and utilization of long-range dependencies while preserving the model’s lightweight nature, an essential factor for object detection tasks. The SCSA module efficiently combines the strengths of both channel and spatial attention, maximizing the use of multi-semantic information to enhance performance in visual tasks. Building on the strengths of both modules, ISAT, which integrates them efficiently, suppresses less important features in both the channel and spatial dimensions. This enables ISAT to fully leverage semantic and long-range features, dynamically adjust attention to features at various scales, and adaptively optimize detection performance for various targets, thereby enhancing its ability to detect objects of various scales and morphologies in complex environments. The architecture of ISAT is illustrated in **Figure 6**.

The C2f-IS module consists of three bottleneck blocks and two ConV blocks, as shown in **Figure 3**. Within the bottlenecks, the lightweight ISAT attention modules are incorporated to enhance the feature of important regions while suppressing background noise. This significantly strengthens the network’s ability to extract essential features. The C2f-IS module performs feature extraction across multiple levels of the network, enabling the model to capture information at different scales and effectively fuse features from feature maps with various resolutions. This enhances the model’s ability to perceive objects of diverse sizes, particularly small objects. By integrating multi-level feature extraction and scale-adaptive mechanisms, the C2f-IS module enhances the model’s robustness and accuracy when processing targets with substantial scale variations. This is particularly advantageous for apple flower detection in this study, given the significant scale difference between apple flowers and buds. The design of this module plays a key role in improving the accuracy of flower detection.

#### 2.2.3 Loss function of YO-AFD

The loss function of YO-AFD involves two branches, the classification branch and the regression branch. In the classification branch, Binary Cross-Entropy (BCE) loss was employed to calculate the classification loss. In regression branch, Distribution Focal Loss (DFL) and Focaler IoU (FIoU) (Zhang and Zhang, 2024) were used to calculate the regression loss. Both BCE loss and DFL loss in YO-AFD remained consistent with those of the baseline YOLOv8. To further improve the performance of the network, the CIoU was replaced with FIoU in the baseline YOLOv8.

FIoU reconstructs the IoU loss utilizing a linear interval mapping approach, which enables it to focus on different regression samples effectively. FIoU analyses the effect of the distribution of difficult and simple samples in bounding box regression on the regression results, further improving the detection performance in detection tasks. The definition of FIoU is shown in Formula 1.


(1) $$\text{FIoU}=\{\begin{array}{ll} 0, & \text{ IoU}<d\\ \frac{\text{IoU}-d}{u-d} & \;d\leq \text{IoU}\leq u\\ 1 & \text{ IoU}>u \end{array}$$

Where, IoU is the original IoU value, and [*d*, *u*]∈[0,1]. By adjusting the value of d and *u*, FIoU can focus on different regression samples. FIoU loss can be defined as Formula 1.


(2) $$L_{\text{FIoU}}=1-\text{FIoU}$$

*L_FIoU_* adjusts the loss value according to the value of IoU. Formulas 2, 3 reveal that *L_FIoU_* is 1 when IoU below the threshold *d*, and *L_FIoU_* is 0 when IoU exceeds the threshold *u*. For IoU value falling between thresholds *d* and *u*, the loss function adopts a linear decreasing trend, rendering it responsive to IoU within a specific range. This allows the loss function to prioritize samples with moderate overlap between predicted and true bounding boxes. Such an approach encourages the model to extract features from samples of moderate difficulty rather than exclusively focusing on the easiest or most challenging ones. In this study, apple flowers present complexities like severe occlusion and high density. These challenges can impede the performance of the original CIoU Loss. Compared to CIoU, FIoU proves more suitable for apple flower detection tasks, offering improved adaptability to address challenges such as severe occlusion and high density.

### 2.3 Network training

The experiments were conducted on a hardware platform featuring an Intel Core i9-11900H @ 2.5GHz CPU, 32GB of memory, and an NVIDIA GeForce RTX 3080 GPU with 16GB of memory. The framework for deep learning was PyTorch 2.2.2 and Compute Unified DeviceArchitecture (CUDA) 11.8. The networks were trained on Windows 10 operating system, and Python 3.8 was used to train and test the apple flower detection network. The hyperparameters used for the proposed network are shown in **Table 2**. For these hyperparameters, the batch size was set according to the memory capacity of hardware platform, while the remaining hyperparameters were set to the default values of the YOLO model, as they have been extensively validated and optimized by the original authors for a wide range of object detection tasks.

**Table 2:** Hyperparameters during training.

Hyperparameters	value
learning rate	0.01
batch size	16
momentum	0.937
weight decay	0.0005
workers	8
iterations	200 epochs

### 2.4 Evaluation criteria

For apple flower detection conducted in this study, it is essential to comprehensively evaluate detection performance. Therefore, parameters including precision, recall, F1 score, and mAP50 were utilized to evaluate the performance. The four parameters can be calculated by Equations 3-6. Higher values of these parameters indicate better detection results.


(3) precision=TP/(TP+FP)×100%

(4) recall=TP/(TP+FN)×100%

(5) F1score=2×precision×recall/(precision+recall)

(6) $$mAP=\frac{1}{n}\sum_{k=1}^{n}AP_{k}$$

Where TP, FP, FN represent true positive, false positive, and false negative, respectively. AP is the average precision of *k*-th class.

To evaluate the impact of class imbalance in the dataset on our method, we further assessed our approach using AUC-ROC (Area Under the Receiver Operating Characteristic Curve). The calculation of AUC-ROC involves dividing the area under the ROC curve into multiple trapezoids, calculating the area of each trapezoid, and then summing them. The formula for this calculation is presented in Equation 7.


(7) $$\text{AUC}-\text{ROC}=\sum_{i=1}^{n-1}\frac{TPR_{i}+TPR_{i+1}}{2}(FPR_{i+1}-FPR_{i})$$

Where, *n* is the number of points on the ROC curve. *TPR_i_* and *FPR*_i_ are the True Positive Rate (*TPR*) and False Positive Rate (*FPR*) at the *i*-th point, respectively.

## 3 Results

### 3.1 Apple flowers detection results

**Figure 7:**
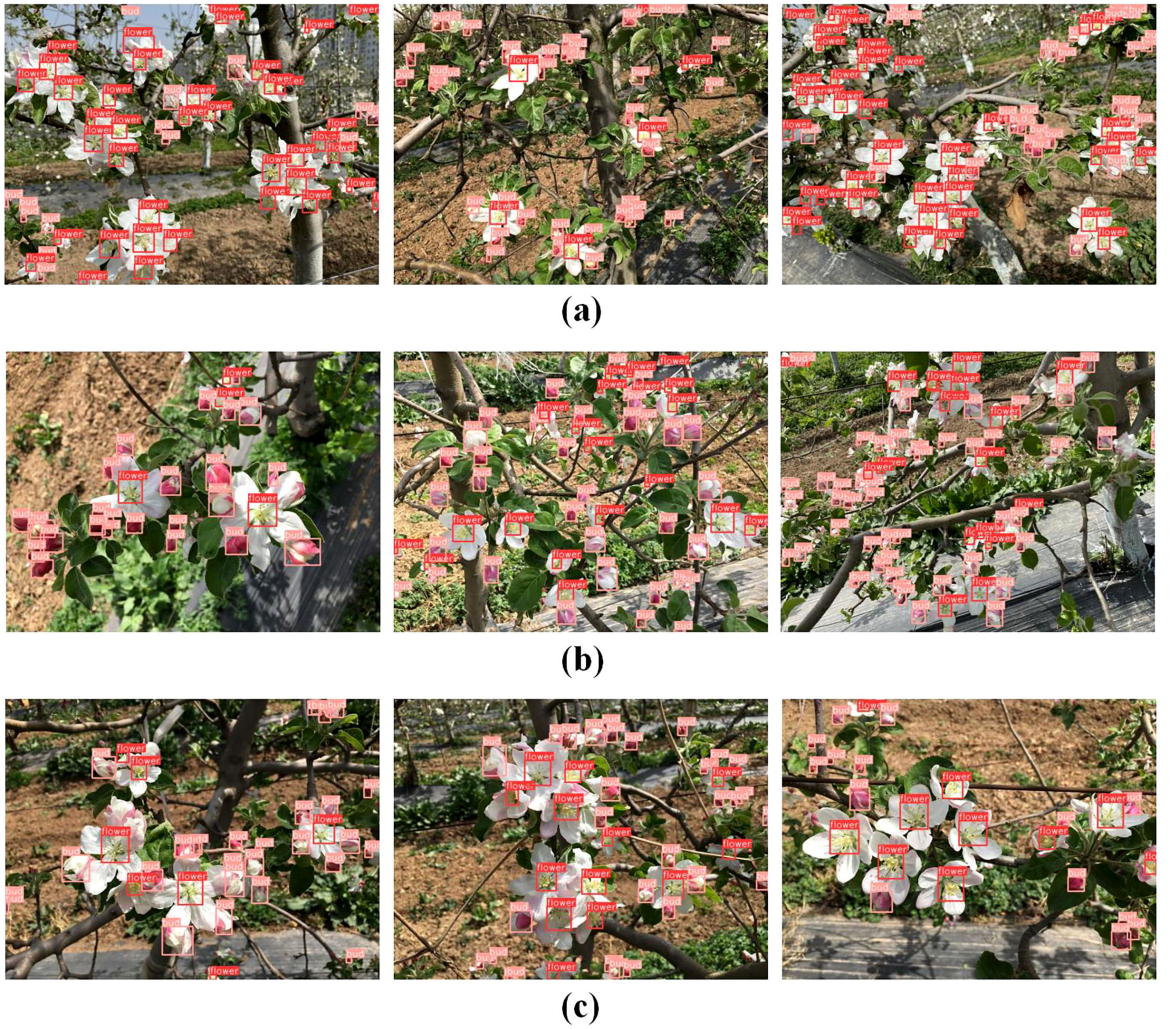
Detection of apple flowers. **(a)** Detection results of images captured under strong lighting and direct sunlight conditions; **(b)** Detection results of images captured under backlight conditions; **(c)** Detection results of images captured under uneven illumination conditions.

To evaluate the performance of the proposed YO-AFD apple flower detection method, a test set containing 420 apple flower images was used to conduct the experiment. The dataset contained a total of 6,210 apple flowers, including 2,208 apple flowers and 4,002 flower buds. The overall detection precision, recall, F1, mAP50 and mAP50-95 were 89.4%, 87.9%, 88.6%, 94.1% and 55.3%, respectively. Examples of the detection results are shown in **Figure 7**, and the detailed detection metrics are summarized in **Tables 3**, **4**.

**Table 3:** Detection results of apple flowers.

Class	Images	Instances	Precision /%	Recall /%	F1 /%	mAP50 /%	mAP50-95 /%
all	420	6210	89.4	87.9	88.6	94.1	55.3
apple flower	420	2208	91.8	90.5	91.1	95.5	51.9
flower bud	420	4002	87.0	85.2	86.1	92.7	58.7

**Table 4:** Detection results of apple flowers under different illumination conditions.

Illumination conditions	Images	Instances	Precision /%	Recall /%	F1 /%	mAP50 /%	mAP50-95 /%
Direct sunlight	231	3608	88.2	88.4	88.3	93.8	54.0
Backlight	189	2586	91.1	87.0	89.0	94.4	57.2
Uneven illumination	235	3681	89.1	88.0	88.5	94.1	55.4

The results presented in **Tables 3**, **4** and **Figure 7** clearly demonstrate the ability of the proposed method to process images captured under a variety of conditions. The method performs well for images taken in bright, sunny conditions with strong illumination (**Figure 7a**) as well as those with uneven lighting (**Figure 7c**). Furthermore, it shows effective detection results under both direct sunlight (**Figure 7a**) and backlight conditions (**Figure 7b**). Specifically, the method achieved precision, recall, F1 score, mAP50, and mAP50-95 of 91.8%, 90.5%, 91.1%, 95.5%, and 51.9%, respectively, for detecting apple flowers across these diverse conditions. For apple flower buds, these metrics were 87.0%, 85.2%, 86.1%, 92.7%, and 58.7%, respectively. To further evaluate the effectiveness of our method, we analyzed the detection results of the YO-AFD method under different illumination conditions, as shown in **Table 4**. For images with direct sunlight and strong illumination, the F1 score, mAP50, and mAP50-95 were 88.3%, 93.8%, and 54.0%, respectively. For images with backlight illumination, these metrics were 89.0%, 94.4%, and 57.2%, respectively. For images with uneven illumination, the metrics were 88.5%, 94.1%, and 55.4%, respectively. The flower detection results were slightly poorer for images with strong illumination due to the formation of shadows on the flower surface under intense lighting, which compromised detection accuracy. In contrast, flower detection performed best in images with even backlight illumination, as fewer complex factors influenced the detection process.

As shown in **Table 2**, the ratio of flowers to buds in the dataset was approximately 1:2. This suggests a degree of class imbalance, though it is not considered highly imbalanced overall. To evaluate the impact of class imbalance in the dataset on our method, we further assessed our approach using AUC-ROC. AUC-ROC provide a comprehensive evaluation of the model’s performance across different decision thresholds, making it particularly robust in scenarios where class distributions are not perfectly balanced. Our method achieved an AUC-ROC of 0.89 on the test set, demonstrating strong performance in effectively distinguishing between flowers, buds, and the background, thereby enabling accurate detection.

### 3.2 Comparison with other detection methods

To conduct a comprehensive performance analysis, the proposed method was compared with several state-of-the-art models, including Faster R-CNN, Dynamic R-CNN (Zhang et al., 2020), YOLOv5n, YOLOv8n, and YOLOv11n, the latest version of the YOLO series. The same training set, validation set and test set were used to train and evaluate the six networks. Additionally, identical hyperparameters (shown in **Table 2**) were applied when training the YOLO series models. The comparison results for all six methods are presented in **Table 5**.

**Table 5:** Comparison of six methods.

Networks	Precision /%	Recall /%	F1/%	mAP50 /%	mAP50-95 /%	Detection Speed /ms	Model Size /MB	Training Time /h
Faster R-CNN (ResNet-50)	77.8	50.8	61.5	60.5	37.4	35.9	321.0	2.84
Dynamic R-CNN (ResNet-50)	75.0	50.2	60.1	57.7	35.9	35.5	319.0	4.33
YOLOv5n	87.6	84.7	86.1	91.3	51.1	**3.7**	**3.9**	0.59
YOLOv8n	88.2	85.2	86.7	92.1	53.0	4.9	6.2	0.58
YOLOv11n	87.4	85.9	86.6	92.0	52.9	**3.7**	5.5	0.32
YO-AFD	**89.4**	**87.9**	**88.6**	**94.1**	**55.3**	5.3	6.5	0.50

Bold text denotes the optimal results.

From the results shown in **Table 5**, it is evident that the YO-AFD method proposed in this study outperformed other methods in terms of accuracy across key evaluation metrics, including precision, recall, F1 score, and mAP. Specifically, the precision of YO-AFD (89.4%) surpassed that of the other methods by 11.6%, 14.4%, 1.8%, 1.2%, and 2.0% compared to Faster R-CNN, Dynamic R-CNN, YOLOv5n, YOLOv8n, and YOLOv11n, respectively. Its recall of 87.9% outperformed the other methods by 37.1%, 37.9%, 3.2%, 2.7%, and 2.0%, respectively. The F1 score of 88.6% for YO-AFD demonstrated significant improvements over Faster R-CNN, Dynamic R-CNN, YOLOv5n, YOLOv8n, and YOLOv11n, with improvements of 27.1%, 28.5%, 2.5%, 1.9%, and 2.0%, respectively. Additionally, the mAP50 (94.1%) and mAP50-95 (55.3%) of YO-AFD showed substantial improvements over all other methods. The detection results in **Table 5** highlight the superior performance of the proposed YO-AFD method in accurately detecting apple flowers. Within the YOLO series, YOLOv8n exhibited higher F1, mAP50, and mAP50-95 values compared to YOLOv5n and YOLOv11n. Building on its superior performance, we conducted our experiments using YOLOv8n as the foundation and subsequently proposed the YO-AFD method for more accurate apple flower detection.

Although the detection speed of YO-AFD (5.3 ms per image) was slightly slower than that of YOLOv5n, YOLOv8n, and YOLOv11n, it maintained good real-time performance when compared to Faster R-CNN and Dynamic R-CNN. Moreover, with a model size of 6.5 MB, YO-AFD remained relatively lightweight compared to Faster R-CNN and Dynamic R-CNN, despite its slightly larger size compared to YOLOv5n, YOLOv8n, and YOLOv11n. In summary, the YO-AFD method significantly enhanced apple flower detection accuracy while maintaining a lightweight model and real-time performance.

As shown in **Table 5**, Faster R-CNN and Dynamic R-CNN exhibit recall rates of 50.8% and 50.2%, respectively, which were significantly lower than those of the other methods. This discrepancy can be attributed to the substantial variation in scale among the apple flowers and buds within the images, which poses challenges for methods that rely on region proposal networks (RPNs), such as Faster R-CNN and Dynamic R-CNN, to accurately detect objects. In contrast, the proposed YO-AFD model integrated the enhanced attention module ISAT into the C2f, allowing the network to better capture the features of apple flowers and effectively focus on targets of various scales and morphologies. This adaptive improvement enabled the YO-AFD model to process the diverse scale distribution present in the images, resulting in enhanced precision and recall, and ultimately, superior detection performance.

## 4 Discussion

### 4.1 Ablation experiments

As discussed earlier, the YO-AFD apple flower detection method proposed in this study is enhanced by incorporating the ISAT module into the C2f of the network’s neck and employing FIoU for loss calculation. To validate the effectiveness of each enhancement, ablation experiments were conducted. All methods were trained and evaluated using the same dataset, and the experimental results are summarized in **Table 6**.

**Table 6:** Ablation experiment.

Model	C2f-IS	FIoU	F1 /%	mAP50 /%	mAP50-95/%	Parameters /×10^6^	GFlOPs	Detection speed /ms	Model size /MB	Training Time /h
YOLOv8n	×	×	86.7	92.1	52.9	3.0	8.2	4.9	6.2	0.58
YOLOv8n-IS	✓	×	87.2	93.6	54.0	3.1	8.4	5.0	6.5	0.57
YOLOv8n-F	×	✓	87.6	93.3	54.2	3.0	8.2	4.2	6.2	0.45
YO-AFD	✓	✓	**88.6**	**94.1**	**55.3**	3.1	8.4	5.3	6.5	0.50

GFLOPs (Giga Floating Point Operations Per Second) refers to the amount of floating-point operations performed during a forward propagation of a model. It is a commonly used metric to measure the computational complexity of a model.

Bold text denotes the optimal results.

**Table 6** shows that among all the methods, the proposed YO-AFD achieved the highest F1 score and mAP. Compared to the baseline YOLOv8n, the F1 score improvements for YOLOv8n-IS, YOLOv8n-F, and YO-AFD were 0.5%, 0.9%, and 1.9%, respectively. Similarly, the mAP50 improvements for these methods were 1.5%, 1.2%, and 2.0%, respectively. To provide a more rigorous evaluation, the mAP50-95 metric was also used to evaluate the methods. The mAP50-95 values for YOLOv8n-IS, YOLOv8n-F, and YO-AFD increased by 2.1%, 2.1%, and 4.3%, respectively, compared to the baseline YOLOv8n model. Notably, each enhancement led to improvements in detection accuracy without causing a significant increase in parameters, computational complexity, model size, or a reduction in detection speed.

The mAP of YOLOv8n-IS, achieved by incorporating the C2f-IS module into the neck of the baseline YOLOv8n, showed significant improvements. For YOLOv8n-F, which employed FIoU to calculate IoU loss, the mAP50-95 also exhibited a notable enhancement. Given these results, fusing both modules into YOLOv8n was expected to further boost model performance. As shown in **Table 6**, integrating these two modules into YOLOv8n led to further improvements in both the F1 score and mAP. The proposed YO-AFD method outperformed all other methods, achieving the highest F1 score and mAP, thereby demonstrating superior detection performance.

The analysis above demonstrates that, although integrating YOLOv8n with the two modules resulted in an increase in model size and a decrease in detection speed, both the F1 score and mAP showed notable improvements. This indicated that the integration of the C2f-IS module and the use of FIoU for IoU loss calculation contributed positively to enhancing the performance of the network model.

### 4.2 Effect of fusion of attention modules

To evaluate the effectiveness of the proposed ISAT attention modules, the YO-AFD method was compared with two variants of YOLOv8n: YOLOv8n integrating the IRMB and FIoU modules (YOLOv8n-IRMB) and YOLOv8n integrating the SCSA and FIoU modules (YOLOv8n-SCSA). The comparison results are summarized in **Table 7**. The results indicate that the proposed YO-AFD method achieved the highest F1 score and mAP, demonstrating superior accuracy in detecting apple flowers. Although the mAP50-95 of the YOLOv8n-IRMB model was high, its computational complexity was substantial. In comparison, YOLOv8n-SCSA detected apple flowers with higher accuracy, fewer parameters, lower computational complexity, and a smaller model size. The fusion of the two modules into the new attention mechanism further improved both the F1 score and mAP, while maintaining a similar model size and parameter count. Notably, despite an increase in network layers, the computational complexity was significantly reduced compared to YOLOv8n-IRMB. Although detection speed was slightly slower than that of YOLOv8n-IRMB and YOLOv8n-SCSA, the detection accuracy showed significant improvement.

**Table 7:** Comparison of different attention modules.

Model	F1 /%	mAP50 /%	mAP50-95/%	layers	Parameters /×10^6^	GFlOPs	Detection speed /ms	Model size /MB
YOLOv8n-IRMB	88.0	93.6	54.0	330	3.1	11.3	4.1	6.5
YOLOv8n-SCSA	88.3	93.7	54.6	290	3.0	8.2	4.3	6.3
YO-AFD	**88.6**	**94.1**	**55.3**	394	3.1	8.4	5.3	6.5

Bold text denotes the optimal results.

### 4.3 Effect of integrating attention modules at different addition positions

**Figure 8:**
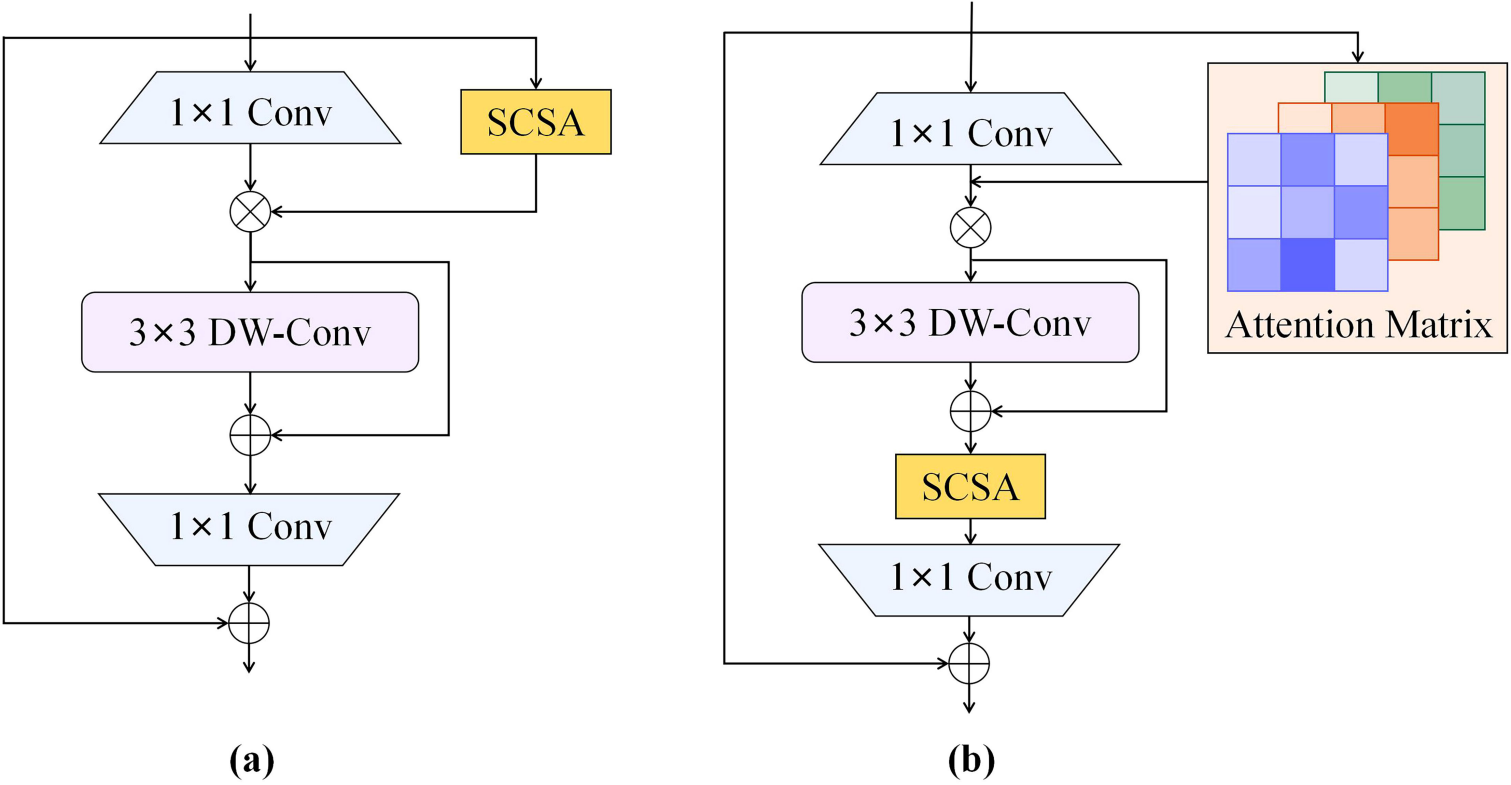
Attention modules with different fusion strategies. **(a)** Fusion method of ISAT*; **(b)** Fusion method of ISAT.

To evaluate the effectiveness of the proposed ISAT attention module, the SCSA module was integrated into two different positions of the IRMB, as depicted in **Figure 8**, for attention mechanism fusion. The performance of the models was then compared, with the results presented in **Table 8**. The comparison revealed a notable variation in performance depending on the position of the integration. In **Figure 8a**, the SCSA module was used to replace the original attention matrix calculation block within the IRMB to generate the attention matrix. However, the performance of this configuration was suboptimal. In contrast, **Figure 8b** illustrates another integration of the SCSA module within the IRMB, which generated the ISAT attention module. The ISAT module preserved the inverted residual structure while effectively combining both channel and spatial attention mechanisms. This design allowed ISAT to leverage multi-semantic features, enhancing the ability to extract long-range dependencies. Furthermore, ISAT was inserted into the C2f module of the network’s neck, where it improved the model’s ability to focus on multi-scale complex features, thereby enhancing overall detection accuracy.

**Table 8:** Comparison of different attention fusion methods.

Model	F1 /%	mAP50 /%	mAP50-95/%	layers	Parameters /×10^6^	GFlOPs	Detection speed /ms	Model size /MB
YOLOv8n-ISAT*	87.9	93.2	53.4	350	3.0	8.3	4.3	6.4
YOLOv8n-ISAT(YO-AFD)	**88.6**	**94.1**	**55.3**	394	3.1	8.4	5.3	6.5

Bold text denotes the optimal results.

## 5 Conclusions

In order to achieve the accurate and rapid intelligent detection of apple flowers in complex natural environments, an apple flower detection method, YO-AFD, based on YOLOv8 and attention mechanism, was proposed in this study. To fully leverage semantic and long-range features while dynamically adjusting attention across various scales, an ISAT attention module that integrated IRMB and SCSA was designed. The designed attention module was then incorporated into the C2f module of the network’s neck to form a C2f-IS module, enhancing the model’s feature extraction capability and multi-scale feature fusion ability. Finally, a regression loss function based on FIoU was employed to calculate the loss of the model, effectively balancing the model’s attention between simple and challenging targets. The test results showed that the YO-AFD model achieved an F1 score of 88.6%, mAP50 of 94.1% and mAP50-95 of 55.3%, respectively. The model size of the YO-AFD was 6.5 MB and the overall processing speed was 5.3 ms per image, satisfying both its lightweight design and real-time detection requirements. Future work will focus on further optimizing the model’s inference speed, as real-time detection is crucial for deployment in agricultural applications. This can be achieved through model pruning, quantization, and knowledge distillation. Additionally, the detection capabilities can be expanded to other fruit flower detection tasks, as well as the identification of flower diseases and pests. These improvements would enhance the versatility of the model and its practical applications in precision agriculture.

## Data Availability

The raw data supporting the conclusions of this article will be made available by the authors, without undue reservation.
